# Supplementary material for: Core–Shell Fe3O4@C Nanoparticles for the Organic Dye Adsorption and Targeted Magneto-Mechanical Destruction of Ehrlich Ascites Carcinoma Cells
Source: Materials (Basel). 2022 Dec 20;16(1):23. doi: 10.3390/ma16010023 (PMC9821792; doi:10.3390/ma16010023)
Supplement: Supplementary file 1 [file materials-16-00023-s001.zip › materials-2053866-supplementary.pdf]

Supplementary Materials

Core–Shell Fe_3O_4@C Nanoparticles for the Organic Dye Adsorption and Targeted Magneto-Mechanical Destruction of Ehrlich Ascites Carcinoma Cells

Oxana S. Ivanova [1,2,*], Irina S. Edelman [1,2], Chun-Rong Lin [3,*], Evgeniy S. Svetlitsky [1], Alexey E. Sokolov [1,2], Kirill A. Lukyanenko [2,4,5], Alexander L. Sukhachev [1], Nikolay P. Shestakov [1], Ying-Zhen Chen [3] and Aleksandr A. Spivakov [3]

1 Kirensky Institute of Physics, Federal Research Center KSC Siberian Branch, Russian Academy of Sciences, Krasnoyarsk 660036, Russia
2 Institute of Engineering Physics and Radioelectronics, Siberian Federal University, Krasnoyarsk 660041, Russia
3 Department of Applied Physics, National Pingtung University, Pingtung City 90003, Taiwan
4 Laboratory of Biomolecular and Medical Technologies, Krasnoyarsk State Medical University Named after Prof. V.F. Voino-Yasenetsky, Krasnoyarsk 660022, Russia
5 Laboratory for Digital Controlled Drugs and Theranostics, Federal Research Center KSC Siberian Branch, Russian Academy of Sciences, Krasnoyarsk 660036, Russia
* Correspondence: osi@iph.krasn.ru (O.S.I.); crlinspin@gmail.com (C.-R.L.)

Table S1. Kinetic parameters of the intra-particle diffusion model parametres for the adsorption of the dyes (C_0 = 60 mg/L for CR and C_0 = 30 mg/L for other dyes) on Fe_3O_4 NPs at 25°C.

Kinetics	Parameters	EoY	CR	MB	RhC
Intraparticle diffusion model	k_1 (mg/(g min$^{0.5}$))	1.25	2.25	0.23	1.04
	C_1 (mg/g)	-0.32	0.05	-0.80	0.13
	R^2	0.96	0.97	0.96	0.98
	k_2 (mg/g min$^{0.5}$)	-0.04	0.23		0.32
	C_2 (mg/g)	8.97	18.30		4.45
	R^2	0.83	0.87		0.96

The values of the coefficient C_1 are negative and close to zero, which implies a small interaction thickness and a minimal diffusion process at this stage.
